# Study of the Interaction of Ti–Zn as a Mixed Oxide at Different pH Values Synthesized by the Sol–Gel Method and Its Antibacterial Properties

**DOI:** 10.3390/nano12121948

**Published:** 2022-06-07

**Authors:** Noé Rodríguez-Barajas, Luis Becerra-Solano, Yanet Karina Gutiérrez-Mercado, Monserrat Macías-Carballo, Claudia M. Gómez, Alejandro Pérez-Larios

**Affiliations:** 1 Centro Universitario de los Altos, Laboratorio de Investigación en Nanomateriales, Agua y Energía, Departamento de Ingeniería, Universidad de Guadalajara, Av. Rafael Casillas Aceves 1200, Tepatitlán de Morelos 47600, Mexico; noe.rbarajas@academicos.udg.mx; 2 Centro Universitario de los Altos, Laboratorio de Biotecnológico de Investigación y Diagnóstico, Departamento de Clínicas, División de Ciencias Biomédicas, Universidad de Guadalajara, Av. Rafael Casillas Aceves 1200, Tepatitlán de Morelos 47600, Mexico; luis.becerra@cualtos.udg.mx (L.B.-S.); yanet.gutierrez@academicos.udg.mx (Y.K.G.-M.); monserrat.macias@cualtos.udg.mx (M.M.-C.); 3 Departamento de Química, División de Ciencias Naturales y Exactas, Campus Guanajuato de la Universidad de Guanajuato, Noria Alta S/N, Col. Noria Alta, Guanajuato 36050, Mexico

**Keywords:** sol–gel method, nanomaterials, mixed oxide, synthesis pH, antibacterial activity

## Abstract

TiO_2_, ZnO, and their combination (TiO_2_–ZnO) at different molar ratios and pH values (Ti–Zn A and B 3:1, 1:1, and 1:3) via the sol–gel method were characterized by SEM, XRD, UV-Vis, and FT-IR. Moreover, antibacterial tests of the nanoparticles were conducted against *Escherichia coli* (*E. coli*), *Salmonella paratyphi* (*S. paratyphi*), *Staphylococcus aureus* (*S. aureus*), and *Listeria monocytogenes* (*L. monocytogenes*). The indirect bandgap of the Ti–Zn binary oxide synthesized in the basic process at molar ratios of 3:1, 1:1, and 1:3 exhibited a higher eV (3.31, 3.30, and 3.19 eV, respectively) compared to pure TiO_2_ (3.2 eV) and synthesized in the acid process (3.22, 3.29, and 3.19 eV at same molar ratio, respectively); in addition, the results of the indirect bandgap were interesting due to a difference found by other authors. Moreover, the sol–gel method promoted the formation of a spherical, semi-sphere, and semi-hexagonal shape (TiO_2_, Ti–Zn 1:1, and Ti–Zn 1:3) with a size ≤ 150 nm synthesized during the acid process, with a crystallite size of ~71, ~12, ~34, and ~21 nm, respectively, while ZnO NPs developed a hexagonal and large size (200–800 nm) under the same synthesis process (acid). Samples were classified as TiO_2_ anatase phase (basic synthesis); however, the presented changes developed in the rutile phase (24% rutile phase) at an acid pH during the synthesis process. Moreover, Ti–Zn maintained the anatase phase even with a molar ratio of 1:3. The most interesting assessment was the antibacterial test; the Ti–Zn A (1:3) demonstrated a bacteriostatic effect compared with all treatments except ZnO, which showed a similar effect in dark conditions, and only Gram-positive bacteria were susceptible (*Listeria monocytogenes* > *Staphylococcus aureus*). Therefore, the Ti–Zn characteristic suggests that the results have potential in treating wastewater as well as in pharmaceutical (as drug carriers) and medical applications.

## 1. Introduction

Nanoscience, through nanotechnology applications, has the objective to improve quality of life and solve problems related with health through nanomaterials, which can be used for medical purpose due to antimicrobial capabilities [1]. These nanomaterials, known as nanoparticles (NPs), can act against bacteria strains due to their photocatalytic and antibacterial effects [2]; in addition, antibiotics have been losing certain antibacterial effects due to present specific resistance to drugs that has become a significant health concern [2,3,4]. Therefore, the development of new potential and effective drugs and new treatments requires a concern to resolve antibiotic resistance and NPs could be that solution [5]. There are organic and inorganic NPs and the latter are composed of metal and metallic oxide nanoparticles (MNPs), which have many applications, including materials science, physics, engineering, and chemistry in which they can act as antibacterial agents [6,7]. These inorganic NPs, such as zinc oxide, silver, zeolite, gold, and titanium dioxide, have been studied for their antimicrobial activity [8,9,10]. Titanium dioxide (TiO_2_) has been extensively studied due to its photocatalytic and antibacterial activities under UV light and the ability to form alloys with other metallics oxides (ZnO, CaO, Al_2_O_3_, SiO_2_, and CuO) [4,6,7,8]. Zinc oxide (ZnO) has a photocatalytic antibacterial effect [11] and can inhibit bacterial growth even in dark conditions [12]. The principal optical properties and some characteristics can be modified by pH due to stabilization to avoid agglomeration by van der Waals forces and magnetic potential [13], and can alter the crystal size at high pH values and form cluster shapes showing large NP clusters [14,15] (under hydrothermal method). However, sol–gel is a synthesis method for making hybrid materials and involves hydrolysis and condensation reactions to alkoxides precursors, such as TiO_2_ and ZnO [5,10,12]. Additionally, the sol–gel route as a synthesis method is simple, accessible, easily controllable, and time- and energy-saving [5,12,15]; moreover, this method provides an excellent process to obtain NPs with useful properties [5,15]. Metal oxides can be covered and linked by inorganic and organic structures by the richness of hydroxyls contained on their surface [16,17,18]; additionally, some organic compounds, such as folic acid, may form bonds through carboxyl reactions in the presence of TiO_2_ [19].

TiO_2_ is a widespread antibacterial material used in many applications, including water purification, food, and pharmaceutical industries [7,20], due to its strong photo-oxidation activity depending usually on UV irradiation [1,2,5,18], which is a restricting factor in its potential applications [21]. Moreover, the antibacterial properties are enhanced due to its high surface area [20] and it has been reported that TiO_2_ under UV irradiation exhibited antibacterial effects against *E. coli*, *S. paratyphi*, *S. aureus*, *Klebsiella pneumonia* (*K. pneumonia*), *Shigella flexneri* (*S. flexneri*), *Vibrio cholera* (*V. cholera*), and *Pseudomona aeruginosa* (*P. aeruginosa*) [1,7,22]. However, TiO_2_ have been doped with other materials, such as Zn^2+^, to improve their antibacterial properties, inhibiting bacterial growth under dark and illuminated conditions [12]. Recently, Suo et al. [12] coupled both NPs to design an aerogel nanocomposite of TiO_2_–ZnO and tested it against bacterial strains, demonstrating good antibacterial properties. Furthermore, Siwińska-Stefańska et al. [23] designed a TiO_2_–ZnO by hydrothermal method that exhibited an excellent antibacterial effect with a TiO_2_–ZnO molar ratio of 1:9 compared to 1:3. On the other hand, Zhang et al. [11] showed the great compatibility of ZnO and TiO_2_ in nanostructure compared to microstructure, due to the ability to release Zn^2+^ ions to kill bacteria cells.

In this work, we synthesize a mixed oxide (Ti–Zn) in two pH conditions (acid and basic pH) by the sol–gel method and characterized it by transmission electron microscopy (SEM), X-ray diffraction (XDR), FT-IR, and UV-Vis spectroscopy. In addition, we evaluate the antibacterial effect of Ti–Zn NPs against *E. coli*, *S. paratyphi*, *S. aureus*, and *L. monocytogenes*.

## 2. Materials and Methods

### 2.1. Material Preparation

For the NPs (Ti–Zn), titanium (IV) butoxide and zinc nitrate were used as precursors and were purchased from Sigma-Aldrich Chemical Co., St. Louis, MO, USA. The method employed was sol–gel adding 20 mL distilled water and 43 mL of ethanol mixed at different concentrations of precursor to obtain the molar ratios of 1:3, 1:1, and 3:1 of TiO_2_ and ZnO, respectively (Table 1). The synthesis of NPs was then adjusted to a pH of 2.48 ± 0.20 and 8.28 ± 0.10 using HNO_3_ (0.1 M) and NH_3_·H_2_O (0.1 M), respectively. First, zinc nitrate was heated for 30 min at 15 °C (excepting the pure TiO_2_ NPs). Then, titanium (IV) butoxide was added (44 mL), and the solutions were heated for 1 h at 20 °C and the pH was adjusted by adding HNO_3_ (0.1 M) and NH_3_·H_2_O (0.1 M) dropwise, respectively; the temperature under reflux was increased to 70 °C constantly for 24 h by magnetic stirring to form a gel. Then, the gel was dried at 100 °C for 24 h to eliminate the ethanolic remains. Before calcining (500 °C for five h in a static air atmosphere with a heating rate of 2 °C/min), the solids were ground to obtain a powder. The pure TiO_2_ and ZnO samples were prepared as described above.

### 2.2. Sample Characterization

The morphology of the samples was determined using Scanning Electron Microscopy (SEM) (Tescan, MIRA3 LMU, London, UK) at 20 kV.

X-ray diffraction (XRD; Empyrean, Malvern Panalytical, Almelo, The Netherlands) equipped with Cu Kα radiation (*λ* = 0.154 nm) over an angular range of 10° to 90° was used to observe the crystallinity. In addition, we used Scherrer’s Equation (1) to calculate the crystal size:

(1) $$D=\frac{K\lambda }{\beta \times cos\theta }$$

where *D* is the crystallite size of the catalyst, λ the X-ray wavelength (1.54060 Å), β the full width at half maximum (FWHM) of the diffraction peak, and θ is the diffraction angle; additionally, there was a correction due to a standard error from equipment (0.01°).

The UV-Vis absorption spectra were obtained with a UV-Vis spectrophotometer (Shimadzu UV-2600, Tokyo, Japan). The bandgap energy was calculated using Planck’s Equation (2):

(2) $$\left(\alpha hv\right)=A{\left(hv-E_{g}\right)}^{n}$$

where *α* is the absorption coefficient, h is the Planck constant, v is the frequency of radiation, *A* is the constant, and *n* is a constant of transitions variance, e.g., for the directly allowed transition, *n* = ½, and for the indirectly allowed transition, *n* = 2.

The Fourier transform infrared radiation (FTIR) spectrum of the NPs was obtained using attenuated total reflectance (ATR) with an FTIR (Nicolet iS5, ThermoFisher Scientific, Tokyo, Japan) spectrometer. The spectrum was recorded with 24 scans and 4 cm^−1^ resolutions at a wavelength from 3700 to 400 cm^−1^.

### 2.3. Antibacterial Activity Test

The antibacterial activity of the Ti–Zn was tested against Gram-negative (*Salmonella paratyphi* ATCC 9150, and *Escherichia coli* ATCC 8739) and Gram-positive (*Staphylococcus aureus* ATCC 33862, and *Listeria monocytogenes* ATCC 15313) bacteria through the agar disc diffusion assay (Kirby–Bauer method) [24]. Bacteria strains were grown in Mueller–Hinton broth (21 g/L, pH 7.3 ± 0.1) for 24 h at 37 °C, and then diluted until they reached 1 × 10^6^ CFU/mL, similar to the 0.5 McFarland standard. The assay was carried out on Muller–Hinton agar plates (38 g/L, pH 7.3 ± 0.1) and inoculated with 100 µL (1 × 10^6^ CFU/mL). Then, sterile standard filter paper discs (4 mm in diameter) were impregnated with sterile suspensions of mixed oxide NPs at 12.5, 25, 50, 75, 100, 250, and 500 µg/mL concentrations, and placed on the plates using sterile forceps. Ampicillin (10 µg/mL) were used as positive control and sterile distilled water as negative control [25]. The plates were incubated at 37 °C for 18–24 h. After this time the diameter of the zone inhibition formed was measured in millimeters (mm). The method described above was repeated for each treatment (TiO_2_, ZnO, and Ti–Zn A and B synthesized at the different molar ratios of 3:1, 1:1, and 1:3) and each bacteria.

### 2.4. Statistical Data Analysis

The antibacterial activity for the *L. monocytogenes* and *S. aureus* data was analyzed by a one-way ANOVA/Tukey’s test. Then, it was evaluated by Levene’s test, *p* > 0.05 (shown homogeneous variances), and Shapiro–Wilk W test, *p* > 0.05 (which presented a normal distribution). The data were obtained from three independent experiments and was performed in triplicate and the results express the mean ± standard deviation. Data were analyzed using the Statistica software (v. 10 Statsoft^®^, Tulsa, OK, USA), with a significance level of α = 0.05.

## 3. Results and Discussion

### 3.1. Morphological Characteristics

**Figure 1:**
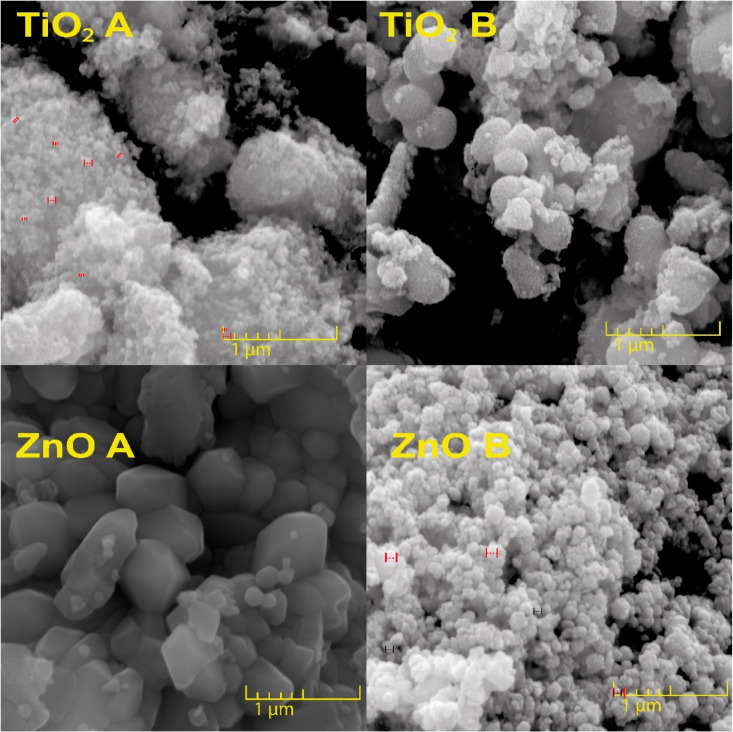
SEM images from the pure TiO_2_ and ZnO nanomaterials synthesized in acid (letter “A”) and basic (letter “B”) pH values.

**Figure 2:**
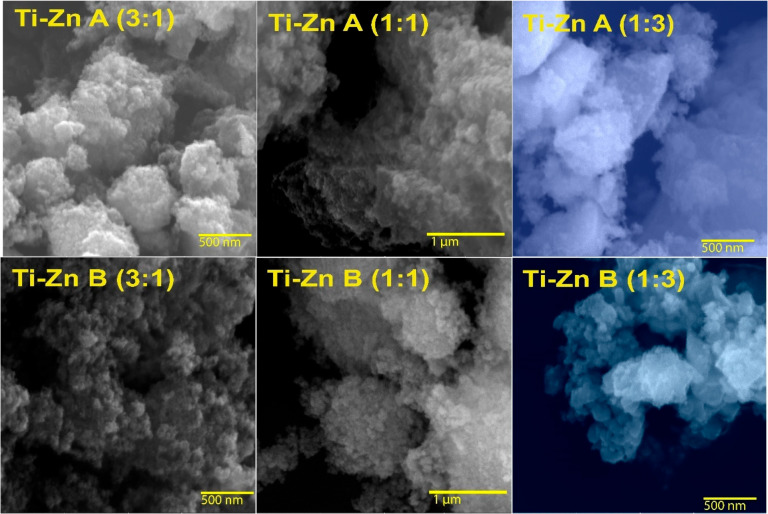
SEM images of Ti–Zn with different molar ratios (Ti–Zn 1:3, Ti–Zn 1:1, and Ti–Zn 3:1) synthesized at different pH values (acid “A” and basic “B”).

The SEM studies of pure TiO_2_ and ZnO, and the combination Ti–Zn are shown in Figure 1 and Figure 2, respectively. Figure 1 shows that the materials exhibited sphere and semi-spheric shapes formed by basic synthesis. It was observed that NPs are in an aggregate state, which takes a spherical agglomeration form with sizes of less than 100 nm in accordance with the measurements obtained with the program ImageJ [7]; this type of agglomeration was reported by Kaur et al. [26], which could be explained by the attraction between TiO_2_–TiO_2_ promoting that kind of spherical agglomeration [26]. However, in an acid synthesis, the NPs conserve their size even in aggregate states without forming spherical shapes (including non-uniform size and superficial agglomeration), which was expected in the sol–gel method of acid synthesis [7,27]. On the other hand, acid-synthesized ZnO NPs acquired a hexagonal form, with a size of 70–120 nm, which is a typical characteristic in ZnO; additionally, SEM showed ZnO tetrapod with a size of 70–800 nm [28], and similar shapes were reported by Venugopal et al. [28]. Moreover, basic-synthesized ZnO NPs exhibited a similar shape with a size of 60–90 nm [29]. Furthermore, the combination of TiO_2_ and ZnO in different molar ratios synthesized at different pH values exhibited a size of 15–50 nm.

Figure 2 shows semi-globular shapes with aggregates (acid- and basic-synthesized Ti–Zn NPs 3:1) and capable of forming aggregate layers composed by NPs with semi-globular shapes, without uniform size due to agglomeration (acid- and basic-synthesized Ti–Zn NPs 1:1). However, in the molar ratio of Ti–Zn 1:3, SEM showed some tubular rounded shapes with a hexagonal profile and a size range of 30–60 nm; these images exhibited a bulk due to agglomeration from the sol–gel method [17].

### 3.2. UV-Vis Analysis

**Figure 3:**
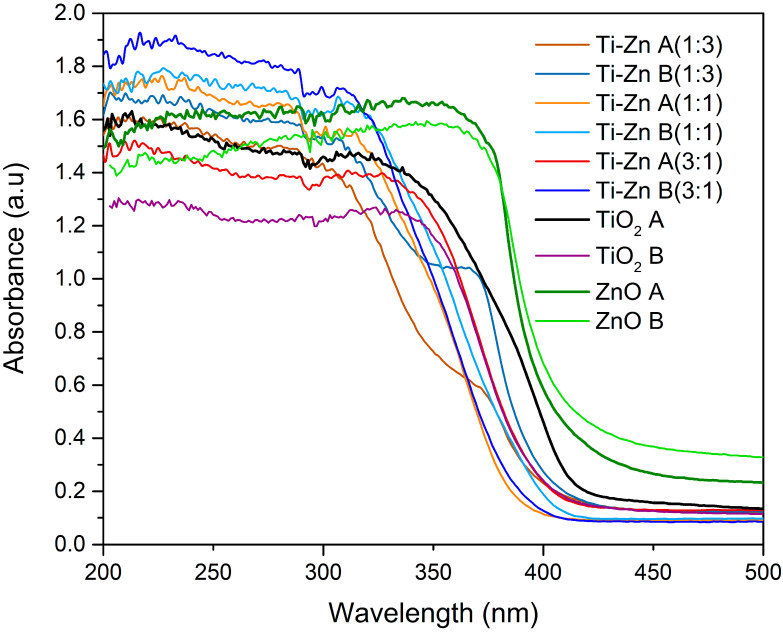
UV-Vis spectra of TiO_2_, ZnO, and Ti–Zn (1:3, 1:1, and 3:1) synthesized at different pH values (acid and basic).

Figure 3 shows the diffuse reflectance spectra obtained for the TiO_2_, ZnO, and the mixed oxide (Ti–Zn). The synthesized material exhibited an absorption starting near to the wavelengths of ~420 and ~390 nm for the TiO_2_ and ZnO structures, respectively, and the similar results of TiO_2_ corresponded with the results by Thandapani et al. [7,30,31], which confirmed that it started to absorb light near ~420 nm [31], and for ZnO, the results showed an absorption peak at ~390 nm, which exhibited a red shift compared to what was reported by Sana et al. [32] and Baruah et al. [33] (375 and 378 nm, respectively). However, Agarwal et al. [34] observed a similar peak absorption (~380 nm) by the hydrothermal method [34]. Additionally, the spectra absorption (TiO_2_) changed due to the molar ratio concentration of ZnO exhibiting a violet shift from ~420 to ~375–(~400) nm, which could be attributed to the ZnO content [35]. Chen et al. [36] reported a blue shift for the TiO_2_ doped with ZnO with other techniques [36]; however, the use of the Tauc plot method for direct bandgap (2) permitted the achievement of the *E_g_* of the pure TiO_2_ acid- and basic- synthetized nanomaterials at 3.05 and 3.16 eV, respectively. For acid- and basic-synthesized ZnO nanomaterials, the *E_g_* values were 3.13 and 3.14 eV, respectively. The result for the acid-synthesized TiO_2_ is similar to that of Nabi et al. [37], which correspond to the anatase phase [37]; the reduction in eV could be due to the state induction of Ti^3+^ in acid with respect to the basic-synthesized material [38] and the smaller particle size of TiO_2_ [39].

**Figure 4:**
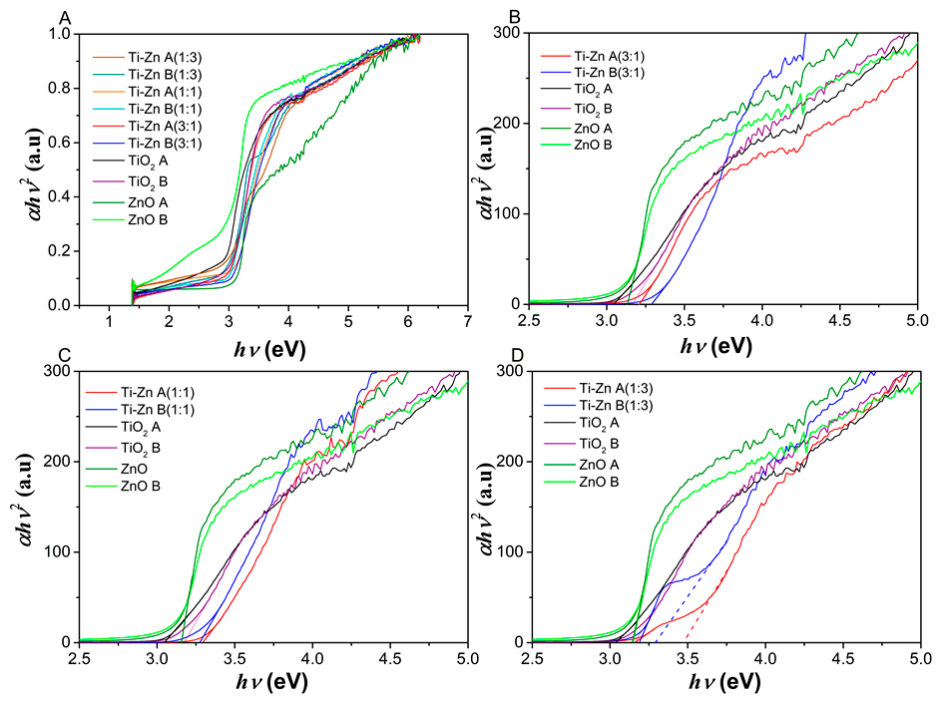
(**A**) Band gap difference between TiO_2_, ZnO, and Ti–Zn (3:1 (**B**), 1:1 (**C**), and 1:3 (**D**), respectively) synthesized at different pH values (acid and basic).

Furthermore, the bandgap of the acid- and basic-synthesized ZnO NPs were 3.13 and 3.14 eV, respectively. These results do not represent considerable differences between them, and the cause could be due to the similar diameter of the ZnO rods; this result is in accord with those of Agarwal et al. [34], who reported that, when ZnO exhibited rods, there is a higher eV compared to when there are flowers shapes (3.19 and 3.0 eV, respectively) [34]. However, the *E_g_* values of Ti–Zn change from 3.29 > 3.22 > 3.16 eV (Ti–Zn A 1:1, 3:1, and 1:3, respectively) and 3.31 > 3.30 > 3.19 eV (Ti–Zn B 3:1, 1:1, and 1:3, respectively), which, contradicts the results observed by Prasannalakshm and Shanmugam [40], who showed an eV decrement that was indirectly proportional to the Zn concentration; however, Pérez-González et al. [41] reported an increment in the *E_g_* values from 3.25 to 3.46 eV due to the increase in pressure in the NPs. In these results, the *E_g_* increment and changes of bandgap could be attributed to the Burstein–Moss (BM) effect, due to filling in of the lower electronic states in the conduction band in heavily doped semiconductors [41] (Figure 4).

### 3.3. Infrared Analysis

**Figure 5:**
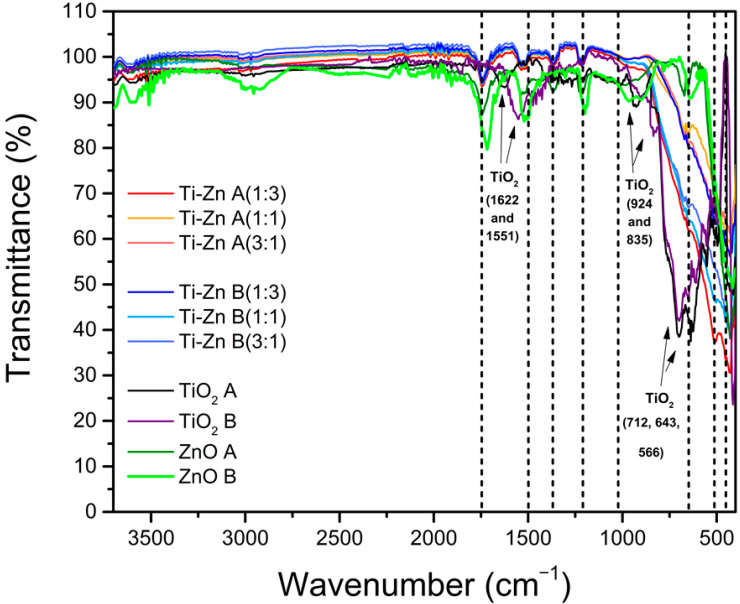
Infrared spectra of TiO_2_, ZnO, and Ti–Zn with different molar ratios (Ti–Zn 1:3, Ti–Zn 1:1, and Ti–Zn 3:1) synthesized at different pH values (acid “A” and basic “B”).

**Figure 6:**
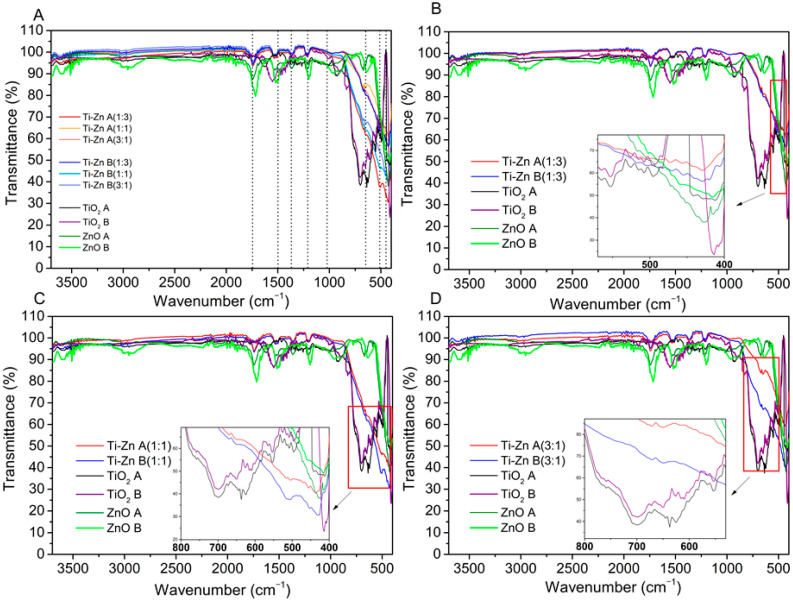
(**A**) Infrared spectra of TiO_2_, ZnO, and Ti–Zn with different molar ratios (Ti–Zn 1:3 (**B**), Ti–Zn 1:1 (**C**), and Ti–Zn 3:1 (**D**)) synthesized at different pH values (acid and basic). The figures inset in each diagram represent an amplification of the red box.

Figure 5 shows the FTIR spectra of TiO_2_, ZnO, and Ti–Zn (1:3, 1:1, and 3:1), with the indicated absorption bands of the anatase phase at the 712, 643, and 566 cm^−1^ stretch vibration bands in the TiO_2_ lattice, as mentioned by Anaya-Esparza et al. [7], and the range of 530–430 cm^−1^ [39,40], which the authors attributed to the Ti–O, Ti–O–C, and Ti–O–Ti bond stretching vibrations also reported in [41]. The absorption bands below 1200 cm^−1^ were due to Ti–O–Ti vibrations. Moreover, the band absorption at 428 cm^−1^ (formed by calcination at 500 °C) could be explained due to the rutile phase formation; this absorption band are in agreement with the results of Haider and Jameel [42], who reported an absorption band at 426.2 cm^−1^ at 900 °C [42]. Furthermore, bands were observed at 3724 cm^−1^ due to the symmetric and asymmetric stretching of water molecules [7]. On the other hand, the ZnO absorption band was observed between 415–430 cm^−1^ and 640–670 cm^−1^ corresponding to Zn–O and C–O bond stretchings, respectively; similar results were reported by Rayyif et al. [43] and Prasanna and Vikayaghavan [44] in ZnO; in addition, the authors reported absorption bands near 1712, 1400, 1156, 1081, and 1017 cm^−1^ [43,44]. Furthermore, Ti–Zn exhibited a similar peak absorption to that of ZnO between 440–425 cm^−1^ due to the Zn–O stretching [42], and the peak absorption in both TiO_2_ synthesized at different pH values showed the particular addition of Ti–Zn evidenced by the peak absorptions at 669, 642 and 540–460 cm^−1^ (Figure 6).

However, the synthesis method in our study used nitric oxide and ammonia hydroxide as pH regulators; the NPs showed absorption bands at 3580 cm^−1^ attributed to the amide group –NHCO– bond stretching vibrations observed by Baldeón-Apaestegui and Hernández-Gorritti [45]. A band was found at 2939 cm^−1^, which was attributed to the stretching vibrations of the –CH_2_ of the aliphatic chain [40]. In addition, the resonances at 1758–1726, 1627, and 1559–1528 cm^−1^ were allocated to the C–C and C–O stretching vibrations, and 1742 cm^−1^ could be attributed to the C=O bond stretching vibration [46]; the frequency present at 1627 cm^−1^ corresponds to the C=O stretching vibrations and, that at 1511 cm^−1^ was attributed to the N–H bending vibrations [47]. Moreover, at 1451 cm^−1^, the vibration corresponded to the CH_2_ bending groups and the peak at 1265 cm^−1^ corresponded to amide III related to the N–H bending [46]. Additionally, a very strong band absorbance around 1049 and 1099 cm^−1^ was observed due to the stretching vibrations of the bond formed between −OH and −NCO (C−O−C) (C−O−C) [7,45,46,48].

### 3.4. X-ray Diffraction

**Figure 7:**
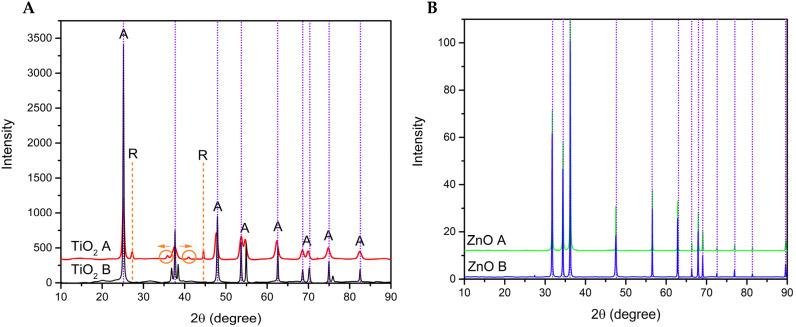
The XRD of TiO_2_ (**A**) where “A” represents the anatase phase and “R” represent the rutile phase, and ZnO (**B**) synthesized at different pH values (acid and basic).

The difractograms of the TiO_2_, ZnO, and Ti–Zn samples are shown in Figure 7. TiO_2_ (Figure 7A) exhibited a different peak formation at both pH values. The principal structure formed by TiO_2_ (basic pH) corresponds to the anatase phase (98%) formed by the sol–gel method and confirmed by XRD analysis with principal peaks at 2θ: 25.16, 36.84, 37.66, 38.42, 47.94, 53.68, 54.91, and 62.57° as well as Miller indices of (101), (103), (004), (112), (200), (105), (211), and (220) planes (JCPDS 01-084-1286), according to the characteristics of the anatase crystalline structure [7,46,47,48,49,50]. In addition, the acid-synthesized TiO_2_ NPs exhibited weak peaks at 27.34 and 44.56°, corresponding to the rutile phase (24%), and blue and red shifts at 35.84 and 40.97°, respectively [49,51,52]. The presence of the rutile phase could be attributed to the higher acidity, which increased the formation of that phase [53]; according to Ibrahim and Sreekatan [49], this phenomenon could occur by a partial charge model due to the positive charge of the hydroxo group, which is not able to condense by spontaneous intramolecular oxolation, avoiding the stabilization of [Ti(OH)_2_(OH_2_)_5_]^2+^ to [TiO(OH_2_)_5_]^2+^. The acid pH level of the oxolation leads to rutile formation, contrary to the basic pH, leading to deoxolation to form the anatase phase [53,54]. ZnO (Figure 7B) demonstrated similar peaks and characteristics at both pH values at 31.76, 34.49, 36.25, 47.53, 56.59, and 62.85°, which correspond to Miller indices of (100), (002), (101), (102), (110), and (103) planes (JCDPS 01-079-2205) [55].

**Figure 8:**
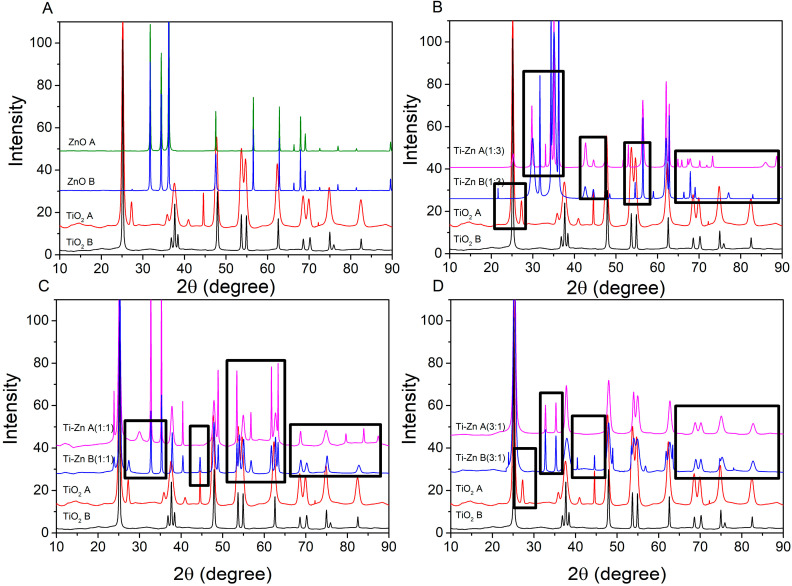
(**A**) Diffractograms TiO_2_, ZnO. And (**B**–**D**) Diffractograms TiO_2_, ZnO, and Ti–Zn with different molar ratios (Ti–Zn 1:3, Ti–Zn 1:1, and Ti–Zn 3:1, respectively) synthesized at different pH values (acid and basic); the principal changes are presented in the black boxes.

In Figure 8B, the principal peak in Ti–Zn A (1:3) maintained the position at 25.2500°, but with a low intensity compared to Ti–Zn B (1:3), which shifted the position to a high angle at 29.9531°; this change could be attributed to a diminution in the Ti/Zn molar ratio and an increment in wurtzite ZnO [36,50] compared to Figure 8A (ZnO as reference). In addition, in Figure 8C the formation of a peak at 21.45° was detected in Ti–Zn B (1:3) and the same peak shifted to a high angle in 2C at 23.85 for Ti–Zn B (1:1) and Ti–Zn A (1:1). This peak was observed by Siwińska-Stefańska et al. [23], which corresponds to the rutile phase (JCPDS No. 21-1279) and ZnTiO_3_ phase (JCPDS No. 26-1500) due to the incorporation of Zn ions into the titania network derived from their similar ionic radii between the rutile and ZnTiO_3_ phases [23]. The peak at 27.42° in the basic-synthesized NP composite corresponded to the rutile phase without alterations [50], but, in the acid-synthesized NP composite, the peak shifted to a high angle of 30.20° with the formation of the brokita phase at 500 °C (JCPDS 02-0514), because there is a change in their surface properties derived from the Zn addition that favors the growth of impurities [51]. However, in Figure 8D Ti–Zn A (3:1) and Ti–Zn B (3:1), both peaks disappeared. Furthermore, the principal peaks observed in Ti–Zn B (1:3) and Ti–Zn A (1:3) corresponding to ZnO exhibited an amorphous morphology due to the space between points 27.9–31.25°, 32.63–38.68° and 29.20–30.59°, 33.85–36.95°, respectively, and an interesting observation is the joining of the characteristic peaks of TiO_2_ and ZnO at those peaks due to this combination of metal oxides, which leads to the inhibiting of the formation of the ZnO crystalline structure by the incorporation of Zn^2+^ that has ionic radii of ca. 60 pm and Ti^4+^ ca. 60.5 pm [17]. These results are in agreement with those of Siwińska-Stefańska et al. [17].

Furthermore, the peak at ~37.67° corresponding to the anatase phase of TiO_2_ is present in all the samples. Moreover, the diffraction patrons of Ti–Zn A and B (1:1) exhibited peaks at 40.38° (only in Ti–Zn A (1:1)) and 44.52°, corresponding to the rutile phase along with the ZnTiO_3_ (JCPDS 26-1500) crystallized sample (TiO_2_–ZnO with a molar ratio of 1:1), reported by Stoyanova et al. [52]; however, the peaks disappeared in Ti–Zn A (3:1), attributed to the high purity of the anatase phase, according to Cano-Casanova et al. [53], and the peaks at 52.54 and 56.45° are characteristic of the ZnTiO_3_ phase (JCPDS No. 14-0033). However, Ti–Zn B (3:1) showed a peak at 55.00°, corresponding to the rutile phase, and exhibited the same peak as Ti–Zn A (3:1) (56.45°) [17]. The peaks at 61.50° and 63.08°, which are characteristics of the ZnTiO_3_ cubic structure, also exhibited an anatase phase and the peaks between 65° and 85° correspond to the ZnO structure, which showed the same cubic structure [17,40,52]; however, although the molar ratio of Ti–Zn 1:3 showed some transition characteristics of the ZnO peaks to TiO_2_ characteristics, the results did not change in the crystal structure of the composite [17,23,39,40].

However, the interactions among the ZnO functionalized with TiO_2_ synthesized at different pH values modify the structure of the composite, evidenced by the formation of new peaks or changing peak position for some samples, principally the main peaks from the pure TiO_2_ and ZnO due to an increase in the molar ratio of ZnO, leading to the formation of Zn_2_TiO_4_ [17].

Furthermore, these changes could be appreciated by the crystallite size obtained by Scherer’s Equation (1), which was used to calculate the crystal size (Table 2).

### 3.5. Antibacterial Activity

Table 3 shows the effect of Ti–Zn at different molar ratios only at a concentration of 250 µg/mL due to doses under that concentration not exhibiting the expected effects on some pathogenic bacteria. The results show statistical differences (*p* < 0.05) between the treatments (acid- and basic-synthesized Ti–Zn) compared with the control drug (ampicillin = 19–28 mm), which exhibited antibacterial activity (10 µg/mL) in all Gram-positive and Gram-negative bacteria. The pristine TiO_2_ did not show an inhibition zone in all the tested bacterial strains compared to the ZnO (at 500 µg) and Ti–Zn A (1:3) treatments, which showed significant antibacterial activities only against *S. aureus* and *L. monocytogenes*. The highest inhibition zone was observed for *L. monocytogenes* (10–11 mm), while the lowest was observed for *S. aureus* (7–8 mm); however, only Ti–Zn A (1:3) at 500 µg/mL showed a high inhibition zone (15–16 and 12–13 mm) against *L. monocytogenes* and *S. aureus*, respectively.

This may be because the antibacterial ability of the NPs doped with mixed oxides, such as TiO_2_ with ZnO, is light-dependent to produce reactive oxygen species (ROS) [17], and in the absence of light, it could release ions of Zn^2+^, promoting changes by inserting these ions and causing membrane resistance to be lost, thus achieving membrane permeability [54]. This directly impacts the penetration of the cell involved by the NPs and the damage to the membrane, given by the binding of the Zn^2+^ ions to the membrane and the catalytic effect that they present by producing hydroxyl radicals producing damage to the bacteria [50], as well as the production of ROS by TiO_2_, since this is involved in the synergy of Zn when these nanomaterials are doped, as reported by Yusuf et al. [50]. Some authors, such as Siwińska-Stefańska et al. [23], have only reported certain antimicrobial-resistant effects against Gram-negative *E. coli*, and observed that Gram-positive bacteria were more susceptible to the activity of NPs with ZnO, which may be due to their cell wall structure and composition, as Gram-positive bacteria contain mainly peptidoglycan (>95%) and teichoic acid. Azam et al. [55] has also reported that NPs based on ZnO or CuO were more effective against Gram-positive bacteria. Additionally, Gram-negative bacteria possess double membranes, making them less susceptible to damage compared to Gram-positive bacteria [23,55].

Some inorganic nanoparticles, such as TiO_2_ and ZnO, present a significant antibacterial effect individually under UV and are well documented. There are certain doubts about the best combination of TiO_2_ and ZnO and their effect without UV. However, research into both materials in combination is limited to the contrast in their bacterial activities.

## 4. Conclusions

The sol–gel method demonstrated an excellent capacity to synthesize TiO_2_ and ZnO NPs; the effect of pH in TiO_2_ showed a change in their size, evidenced by a small nanoparticle in acid pH compared to basic pH, which increased their size. A contrary effect was observed in ZnO, which exhibited the largest particle size in the basic synthesis with a defined hexagonal form compared to the small particles in the basic synthesis, forming agglomeration. The sol–gel method also has greater effectiveness in the synthesized TiO_2_–ZnO binary oxides, which exhibited a lower pH-value-dependent crystallite size due to the smaller crystallite size in all Ti–Zn molar ratios synthesized in acid compared to those that were basic synthesized; at the same time, the molar ratio of 1:3 maintained the crystallinity, and the TiO_2_ still presented the anatase phase. Additionally, the pH could change *E_g_* values, demonstrated by an increment in eV in the Ti–Zn synthesized by basic pH compared to that synthesized in acid pH at the same molar ratio. The TiO_2_–ZnO oxide with a molar ratio of 1:3 synthesized in acid pH showed a slightly antibacterial effect in Gram-positive bacteria at 250 and 500 µg; the most affected bacterium was *S. aureus* with a zone inhibition of 15.33 ± 0.76 at 500 µg compared to the effects produced by ZnO. Furthermore, it is important to note that all experiments were performed in the absence of light due to the effect of light having been documented as possibly being photoactive in promoting antibacterial effects in the NPs; for this reason, the acid-synthesized Ti–Zn (1:3) could have potential in medical applications as an antibacterial agent.

## Figures and Tables

**Table 1:** The pH of the synthesized NPs and *E_g_* values at different molar ratios.

Code	Relation Molar	pH (HNO_3_)	pH (NH_3_·H_2_O)	Direct Bandgap (*E_g_* in eV)		Indirect Bandgap (*E_g_* in eV)	
TiO_2_	1	2.56	8.37	2.77 ^a^	2.82 ^b^	3.02 ^a^	3.20 ^b^
Ti–Zn3	3:1	2.51	8.26	2.83 ^a^	2.92 ^b^	3.22 ^a^	3.31 ^b^
Ti–Zn2	1:1	2.27	8.28	2.86 ^a^	3.03 ^b^	3.29 ^a^	3.30 ^b^
Ti–Zn1	1:3	2.68	8.18	2.87 ^a^	2.94 ^b^	3.16 ^a^	3.19 ^b^
ZnO ^c^	1	2.38 ^c^	8.34	2.96	2.90	3.17	3.15

^a^ Synthesized in acid pH; ^b^ Synthesized in basic pH; ^c^ Results used as the control dopant.

**Table 2:** Size of the crystallite.

Material	Crystal Size (nm)	Principal Peak Position (°2θ)	*d-Spacing* (Å)	(hkl)	a (Å)	c (Å)
**TiO_2_A**	12.26 ± 0.22	25.153	3.5376	[101]	3.85	10.50
**TiO_2_B**	23.87 ± 0.78	25.215	3.5290	[101]	3.78	9.52
**ZnOA**	69.05 ± 5.81	36.216	2.4780	[101]	3.25	5.20
**ZnOB**	70.82 ± 5.50	36.178	2.4808	[101]	3.14	5.21
**Ti–Zn A (3:1)**	17.19 ± 0.51	25.308, 35.288	3.5163, 2.5413	[101] [212]	7.95	6.151
**Ti–Zn B (3:1)**	41.30 ± 3.60	25.278, 35.829	3.5204, 2.5413	[101] [021]	9.93	8.19
**Ti–Zn A (1:1)**	36.34 ± 2.20	25.163, 35.258	3.5362, 2.5434	[101] [300]	7.91	10.93
**Ti–Zn B (1:1)**	34.55 ± 1.57	25.223, 35.265	3.5279, 2.5429	[101] [205]	9.62	5.93
**Ti–Zn A (1:3)**	21.57 ± 0.68	25.156, 36.637	3.5371, 2.4508	[101] [004]	5.94	6.39
**Ti–Zn B (1:3)**	29.71 ± 1.30	29.930, 36.204	2.9829, 2.6039	[220] [302]	5.99	8.42

**Table 3:** Antibacterial activity of the pure TiO_2_ and ZnO, and Ti–Zn (acid- and basic-synthesized).

Treatment	*E. coli* (mm)	*S. paratyphi* (mm)	*S. aureus* (mm)	*L. monocytogenes* (mm)
Ampicillin (C+) ^a^	19.78 ± 1.09	28.56 ± 1.24	24.44 ± 0.88	26.22 ± 1.09
Distilled water (C−)	0 ± 0	0 ± 0	0 ± 0	0 ± 0
TiO_2_	0 ± 0	0 ± 0	0 ± 0	0 ± 0
ZnO	0 ± 0	0 ± 0	7.89 ± 0.60 ^b^	9.22 ± 1.09 ^b^
Ti–Zn A (3:1)	0 ± 0	0 ± 0	0 ± 0	0 ± 0
Ti–Zn B (3:1)	0 ± 0	0 ± 0	0 ± 0	0 ± 0
Ti–Zn A (1:1)	0 ± 0	0 ± 0	0 ± 0	0 ± 0
Ti–Zn B (1:1)	0 ± 0	0 ± 0	0 ± 0	0 ± 0
Ti–Zn A (1:3)	0 ± 0	0 ± 0	7.67 ± 0.58 ^b^	10.17 ± 1.04 ^b^
Ti–Zn B (1:3)	0 ± 0	0 ± 0	0 ± 0	0 ± 0

Source: Antibacterial test of Ti–Zn NPs synthesized at acid and basic pH values against Gram-positive and Gram-negative strains. Data were analyzed by the Kruskal–Wallis test set at *p* < 0.05. ^a^ (10 µg/mL). ^b^ Bacteriostatic activity in accordance with Anaya et al. [2].

## Data Availability

Not applicable.
